# A global analysis of low-complexity regions in the *Trypanosoma brucei* proteome reveals enrichment in the C-terminus of nucleic acid binding proteins providing potential targets of phosphorylation

**DOI:** 10.12688/wellcomeopenres.16286.2

**Published:** 2020-11-18

**Authors:** Mathieu Cayla, Keith R. Matthews, Alasdair C. Ivens

**Affiliations:** 1 Centre for Immunity, Infection and Evolution, Institute for Immunology and Infection Research, School of Biological Sciences, University of Edinburgh, Edinburgh, Scotland, EH9 3JT, UK

**Keywords:** Low-complexity regions (LCRs), proteome, phosphorylation, liquid-liquid phase separation, nucleic acid binding proteins, granules

## Abstract

**Background: **Low-complexity regions (LCRs) on proteins have attracted increasing attention recently due to their role in the assembly of membraneless organelles or granules by liquid-liquid phase separation. Several examples of such granules have been shown to sequester RNA and proteins in an inactive state, providing an important mechanism for dynamic post-transcriptional gene regulation. In trypanosome parasites, post-transcriptional control overwhelmingly dominates gene regulation due to the organisation of their genome into polycistronic transcription units. The purpose of the current study was to generate a substantially more comprehensive genome-wide survey of LCRs on trypanosome proteins than currently available *. *

**Methods: **Using the Shannon’s entropy method, provided in the R package ‘entropy’, we identified LCRs in the proteome of *Trypanosoma brucei*. Our analysis predicts LCRs and their positional enrichment in distinct protein cohorts and superimposes on this a range of post-translational modifications derived from available experimental datasets.

**Results: **We have identified 8162 LCRs present on 4914 proteins, representing 42% of the proteome, placing *Trypanosoma brucei* among the eukaryotes with the highest percentage of LCRs *.* Our results highlight the enrichment of LCRs in the C-terminal region of predicted nucleic acid binding proteins, these acting as favoured sites for potential phosphorylation. Phosphorylation represents 51% of the post-translational modifications present on LCRs compared to 16% on the rest of the proteome.

**Conclusions: **The post-translational modifications of LCRs, and in particular phosphorylation events, could contribute to post-transcriptional gene expression control and the dynamics of protein targeting to membraneless organelles in kinetoplastid parasites.

## Introduction

Prion-like-domains are responsible for the self-aggregation of proteins into amyloid-fibres causing, for example, neuro-degenerative diseases. These domains present lower amino-acid complexity than the surrounding background and are frequently enriched in polar amino acids such as asparagine and glutamine ^1^. Contrasting with these fibres, low-complexity regions (LCRs) can also contribute to biological function, an example being in ribonucleotide binding proteins that assemble dynamic polymers in a hydrogel state, via liquid-liquid phase separation ^2^. The ability of LCRs to influence the liquid-liquid phase separation of proteins, resulting in the formation of membraneless organelles or granules in different cellular compartments, creates a specialised local environment such as the nucleolus or for example P-bodies and stress granules. The latter are responsible for a local sequestration of RNA and proteins in an inactive state ^3^. As a consequence, the analysis of LCRs has developed over the last two decades from a pathogenic curiosity to a new exciting field of research focused on regulatory gene expression operating at the post-transcriptional level.

One group of organisms that show a marked reliance on post-transcriptional regulation of gene expression is kinetoplastid parasites. These include the important tropical pathogens *Trypanosoma cruzi*, *Leishmania* spp and the experimentally tractable African trypanosome, *Trypanosoma brucei*. These organisms transcribe RNAs as part of polycistronic transcription units that are subsequently processed to mRNA. As a result, transcriptional regulation is not a significant contributor to differential gene expression. Rather, genes are regulated through mRNA stability and translation. Several protein factors have been identified that contribute to the stability of mRNAs and their relative translational competencies. When characterised cytologically, it has been observed that some mRNA regulators concentrate into discrete foci under conditions of cellular stress, or during life cycle development. The foci resemble nuclear periphery granules, pole granules, P-bodies and stress granules. Similar to other eukaryotes, these structures are compositionally enriched in nucleotide binding proteins and translation initiation factors ^4^.

By inference from what is known for other model eukaryotes, it is plausible that the aggregation into membraneless structures could be influenced by the presence and/or distribution of LCRs in the protein sequences themselves ^5^. At present, information on predicted LCRs in the *T. brucei* proteome can be obtained from the TriTrypDB genome website as an implementation of the SEG algorithm, which does not account for amino acid usage across the proteome ^6^. These available data were derived using a limited range of parameters, yielding a potentially sub-optimal output in terms of broader applicability or utility ^7^. The goal of the current study was to generate a substantially more comprehensive LCR dataset for the encoded *T. brucei* proteome that would enable us to explore their potential association with distinct protein families or as targets of post-translational modifications. Our analysis provides an enhanced description of LCRs across the trypanosome proteome and highlights their enrichment in the C-terminal region of predicted nucleic acid binding proteins. Moreover, analysis of experimentally determined post-translational modifications on proteins suggests that the LCRs of RNA-binding proteins might be a preferential site of phosphorylation that could contribute to post-transcriptional gene expression control in kinetoplastid parasites.

## Methods

### LCR identification – entropy method

Protein sequences for *Trypanosoma brucei brucei* TREU927/4 were obtained from the TriTrypDB website in fasta format (release 46) ( https://tritrypdb.org/common/downloads/Current_Release/TbruceiTREU927/).

All processing of the sequences was performed in the R/Bioconductor environment using BioStrings ^8^, entropy ^9^, dplyr ^10^, and bedr ^11^ packages.

Briefly, each protein sequence was processed as a series of overlapping windows, with each subsequent window starting one amino acid further towards the carboxy terminal. For each of the full-sized windows, amino acid entropy was calculated using the entropy.plugin() function ^9^. The empirical cumulative distribution function (ecdf) distribution was calculated for all entropy values for the window size, and a threshold value at 0.5% determined. All amino acid sequence windows with entropy values below this threshold were deemed to be part of an LCR. Overlapping LCR regions within the same protein sequence were subsequently merged using the bedr R cran package ^11^.

This process was repeated for a series of amino acid window sizes (10, 20, 30, 40, 50, 60, 75, 100, 150). Once all 0.5% threshold LCR regions had been identified for each of the nine different window sizes, these were in turn merged, using the bedr package, for further analysis.

The R scripts used to perform the analyses are provided (LCR_TREU927_RSCRIPTS.tar.gz, see *Data availability*) ^12^.

### InterPro domain mapping

InterPro domain mapping information was obtained from TriTrypDB (release 46) in tab-delimited text format. Regions of InterPro domain overlapping with the LCR regions were determined using bedtools intersect (v2.23.0).

### Sequence property analysis

Properties of amino acid sequences, including the acid, aliphatic, aromatic, basic, bulkiness, net-charge, hydropathy, length and polarity indices were obtained with the alakazam R package ^13^.

### PTM mapping

Post-translational modification (PTM) mapping information was obtained from available online datasets: phosphorylation during the *T. brucei* (procyclic form) cell cycle ^14^, post-translational modification of *T. brucei* and *T. b. evansi* bloodstream forms ^15^, differential phosphorylation analysis between bloodstream and procyclic stage of *T. brucei* ^16^, phosphorylation in the TbDYRK knock-out strain of *T. brucei* ^17^, phosphorylation events during heat shock ^18^, comparative analysis of lysine acetylation in trypanosomes ^19^, arginine methylation in slender forms of *T. brucei* ^20^, arginine methylation in mitochondria of *T. brucei* ^21^.

### Gene Ontology analysis

The molecular function Gene Ontology analysis was performed on the TriTrypDB website from computed and curated association with a p-value cutoff of 0.01.

### Statistical analysis

Statistical analysis comparing proportions were performed using a z-test for the PTMs analysis and for the comparison of categorical variables, i.e. the location of LCRs, using a Chi-squared test in R.

## Results

### The T. brucei proteome is biased toward some amino acids

**Figure 1.**
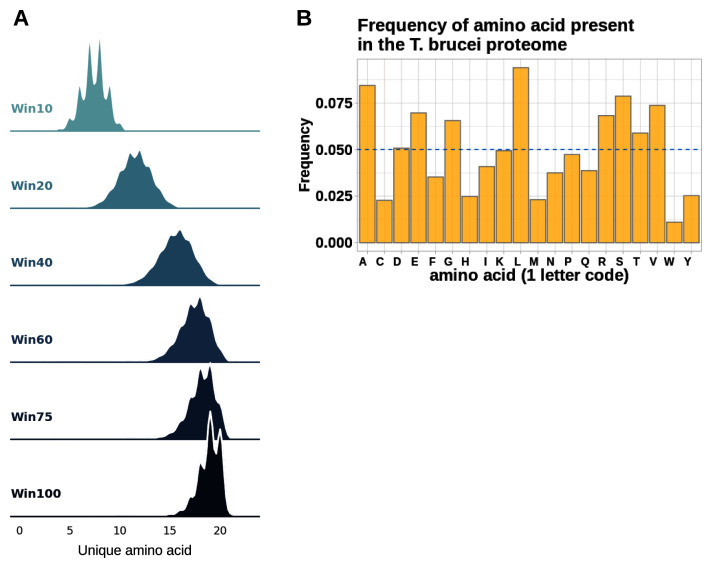
Amino-acid diversity in the *Trypanosoma brucei* proteome. **A**) The *T. brucei* proteome has been processed as a series of different window sizes, ranging from 10 to 150 amino acids, and the distribution of unique amino acids per window visualized; six of the nine window sizes assessed are illustrated. **B**) Frequency of each amino acid in the *T. brucei* proteome. Amino acids are indicated with the one letter code and the 1/20th value represented by the blue dashed line.

The widely used algorithm to identify LCRs, SEG, is based on an analogue measure of the Shannon’s entropy, assuming a uniform probability of representation of each amino-acid ^6^. This also implies that LCRs have to be intrinsically distinct from their surroundings to be detected. Therefore, we initially analysed the *Trypanosoma brucei* proteome to determine if there was evidence for a bias in the representation of particular amino acids. The proteome was processed as a series of amino acid window sizes (10, 20, 30, 40, 50, 60, 75, 100 and 150) and examples of the density of unique amino acids per window represented in Figure 1A. Interestingly, we observed a clear bias towards particular amino acids. Indeed, the mean number of unique amino acids was only 11.51 ± 1.65 with a window of 20 amino acids, 17.34 ± 1.64 unique amino acids were present per window of 60 amino acids, and only for the windows 75 and 100 did we observe the 20 amino acids represented within one window, with a mean of unique amino acids per window of 18.09 ± 1.52 and 18.8 ± 1.34, respectively.

In regards of this apparent bias, we then calculated the relative abundance of the 20 different amino acids and compared them to the expected frequency if all amino acids were equally present (0.05, dashed blue line, Figure 1B). Eight amino acids were over-represented in the proteome of *T. brucei*, including for example alanine, leucine, serine and threonine, whereas five amino acids were present at half the expected frequency: cysteine, histidine, methionine, tryptophan and tyrosine. The other eight amino acids presented an abundance ranging from the expected value (aspartic acid, lysine and proline) to 0.025 ( Figure 1B). These results are similar to those obtained in the study of codon bias usage in a set of highly expressed genes ^22^ and led us to re-visit the LCR prediction for the proteome of *T. brucei,* with a method that takes into account the compositional bias of amino acids in the proteome.

### LCR calling using the Shannon’s entropy method

**Figure 2.**
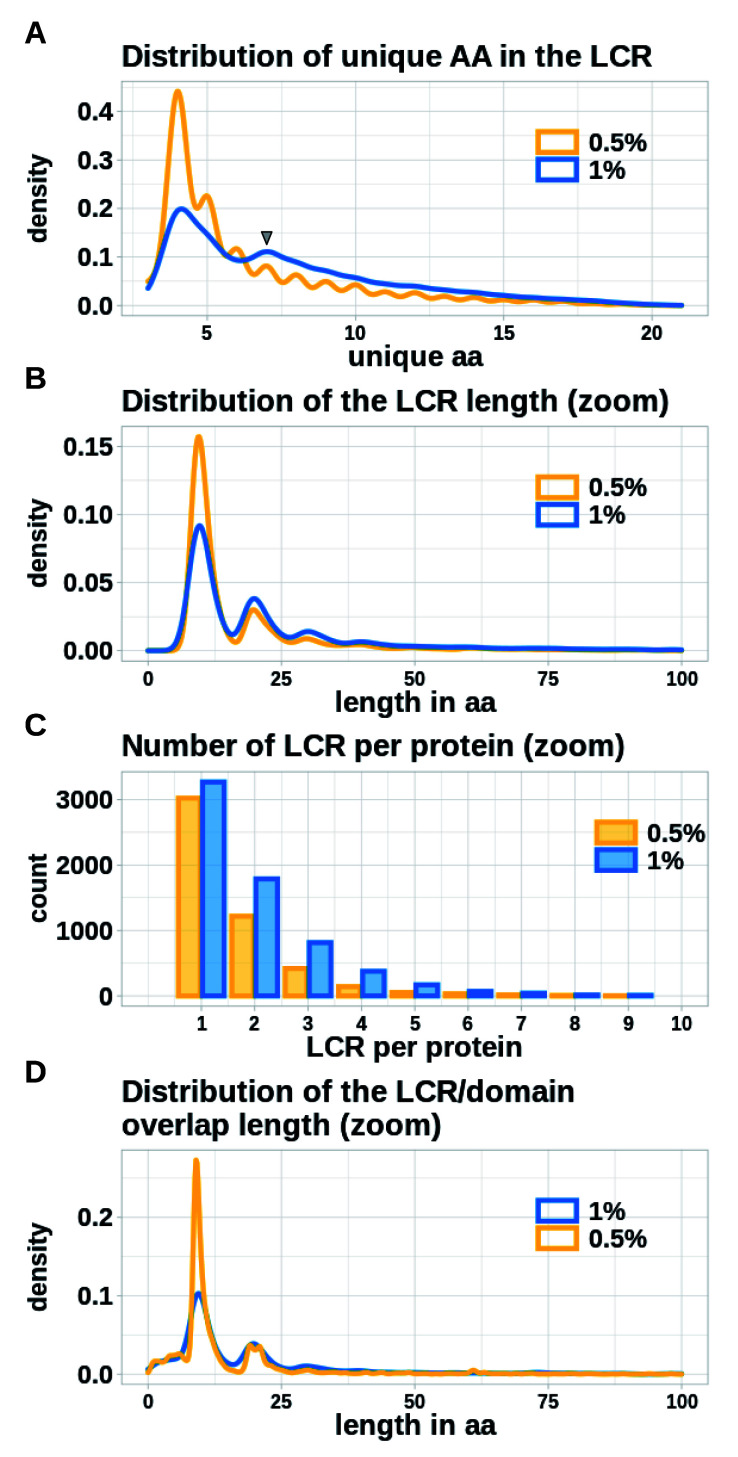
Comparison of the two different empirical cumulative distribution function (ecdf) thresholds, 1% and 0.5%. **A**) Distribution of unique amino acids per low-complexity region (LCR) after merging. The arrowhead indicates the second peak of seven unique amino acids per LCR present with the 1% threshold. **B**) Distribution of the length of the LCRs, zoomed to include only those in the range from 0 to 100 amino acids. **C**) Distribution of the numbers of LCRs per protein, zoomed to include only those in the range from 0 to 10 LCRs per protein. **D**) Analysis of the LCRs identified by the entropy method overlapping with domains identified in the InterPro database. Size distribution of the overlapping regions, zoomed to include those in the range from 0 to 100 amino acids.

To examine the LCRs in the proteome of *T. brucei*, we used the Shannon’s entropy calculation ^23^, a well-accepted methodology to measure complexity in biological sequences. We processed the proteome as a series of amino-acid window sizes ranging from 10 to 150 amino acids, with each subsequent window being one amino acid further towards the carboxy terminal. As indicated by Battistuzzi *et al.* ^7^, for the SEG algorithm, the initial parameters chosen for the threshold of selection of the LCRs determine the final identification. The ecdf was calculated for all entropy values for the window size, and different thresholds, from 0.5 to 5 %, were plotted on each of the cumulative curves (Figures S1 and S2, *Extended data* ^24^). As described in Coletta *et al.* ^25^, we visually inspected the thresholds to subjectively select the portion under the curve where the flat tail is located. Two stringent entropy thresholds were first selected, i.e. 0.5% and 1%, below which a region was deemed to be a putative LCR. As described in the ‘Methods’, overlapping LCRs within the same protein sequence were subsequently merged among each window size and between the different windows as well. The final LCRs obtained were then compared for the two thresholds. We were able to identify 12933 or 8162 unique LCRs on 6579 or 4914 unique proteins (59% or 43.8% of the proteome) using the 1% or 0.5% thresholds, respectively. The distribution of unique amino acids per LCR ( Figure 2A) indicates that for both thresholds, LCRs are mainly composed of four to five different amino acids. There is a second peak at seven amino acids with the 1% threshold (grey arrowhead on Figure 2A).

**Figure 3.**
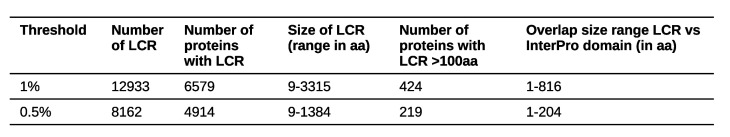
Values for different low-complexity region (LCR) parameters obtained from the 1% and 0.5% analysis threshold.

LCRs identified with the 1% threshold ranged in size from nine to 3315 amino acids, whereas with the 0.5% threshold, LCR regions ranged from nine to 1384 amino acids. Of the 6579 or 4914 proteins containing predicted LCRs, relatively few, 424 or 219, were longer than 100 amino acids, using the 1% or 0.5% thresholds, respectively. When the 0.5% threshold was applied, ( Figure 2B; Figure 3) there was a global reduction of the size of the LCRs, with a relative enrichment of LCRs with a size ranging from nine to 18 amino acids.

Next, we compared the number of LCRs per protein using the two thresholds. Figure 2C indicates a minor reduction in the number of LCRs per protein with the 0.5% threshold compared to 1%, likely due to the fewer number of LCRs identified with this more stringent threshold ( Figure 3). Finally, we explored the size of the overlapping regions of the LCRs with domains identified in the InterPro database. Overlaps ranged from one to 816 or 204 amino acids, respectively, using the 1% or 0.5% thresholds. Both thresholds presented the same pattern with two peaks, one between ~9 to 12 amino acids overlap and one between ~16 to 19 amino acids overlap ( Figure 2D; Figure 3). We note that there is, however, an over-representation of the first peak with the 0.5% threshold suggesting a reduction of the overlap with this setting.

In conclusion, the more stringent threshold (0.5%) selects for shorter LCRs that are of relatively lower complexity and reduces the size and frequency of overlap with previously identified domains, without significantly affecting the number of LCRs per protein. Therefore, we applied the most stringent 0.5% threshold for the remainder of our analysis.

Previous information available on LCRs on the TriTrypDB website were generated using the SEG algorithm. We therefore identified LCRs using this algorithm to compare the results obtained with the entropy methodology using the 0.5% threshold. We chose three different window sizes of 12, 25 and 45 amino acids, with a complexity threshold of 2-2.2, 3-3.3, 3.4-3.75 as initial parameters, as described in Wotton *et al*. 1994 ^6^. The results indicate that the SEG algorithm is highly dependent on the initial window size parameters, as previously observed ^7^, with the complexity in amino acids and the length of the LCRs varying greatly for each window size (supplement figure S3, *Extended data* ^24^; supplement file 2, *Underlying data* ^26^). A similar distribution of the number of LCR per protein is observed with the different windows and with the entropy methodology. We also note the presence of extremely long LCRs obtained with the SEG methodology. 1433 proteins present LCRs identified with both methodologies with any initial parameters, 2486 proteins are identified with the entropy and at least one parameter of the SEG methods, and 435 proteins are unique to the entropy methodology (supplement figure S3, *Extended data* ^24^; supplement file 2, *Underlying data* ^26^). In conclusion, this analysis indicates that the entropy methodology allows the identification of more diverse LCRs, is not biased by the initial parameters chosen and limits the identification of very long, potentially artefactual, LCRs.

**Figure 4.**
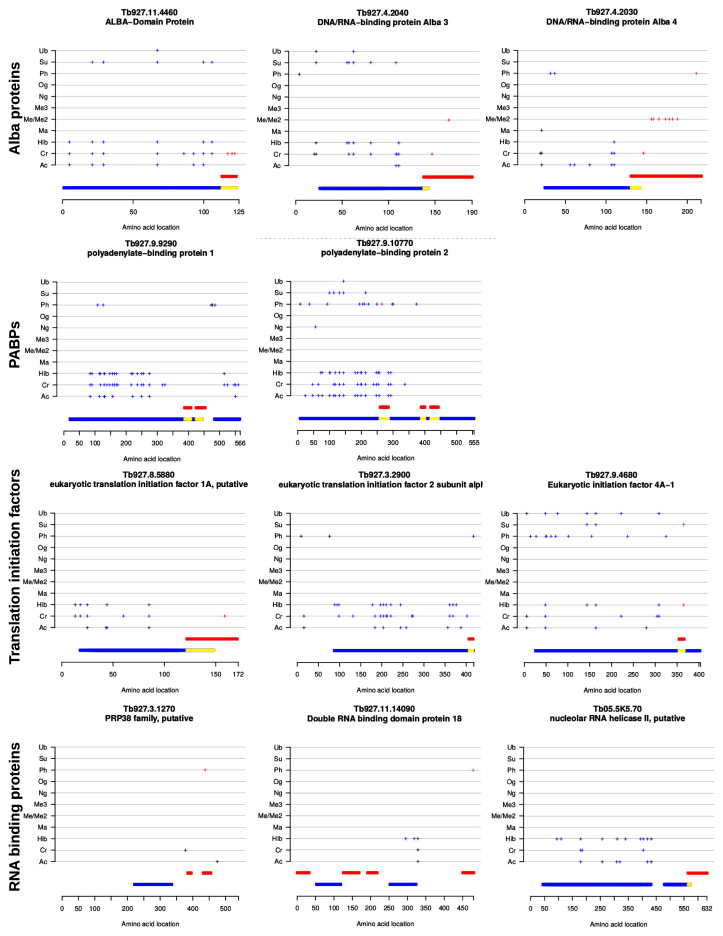
Examples of supplementary file 1 (see *Extended data*) protein pages. Each plot represents a protein (ID and product). The X-axis indicates the protein size in amino acids and on the plot are represented the final combined low-complexity regions (LCRs; in red), the identified InterPro domains (in blue) and the overlap regions between LCR and InterPro domain indicated in yellow. Post-translational modifications (PTMs) identified in experimental analysis by different studies are indicated above by “+” symbol. Each modification is coloured in blue when present in an InterPro domain, in red when present in an LCR or in black when present in neither.

To represent each predicted protein in the proteome, a series of plots was generated for all proteins encoded in the trypanosome genome, excluding variant surface glycoproteins (VSGs; supplement file 1, *Extended data* ^24^), where we indicate the combined final LCR, obtained by the entropy method with the 0.5% threshold, in red, as well as the InterPro domains in blue and the overlapping regions in yellow. Examples of Alba proteins, polyadenylate-binding proteins, translation initiation factors and RNA-binding proteins are presented in Figure 4. In addition, we show the position of the distinct post-translational modifications (PTMs) identified in different published datasets ^14– 17, 19, 20^. The corresponding dataset of the *Trypanosoma brucei* proteome with the start and end position of InterPro domains and identified LCRs can be found in supplement file 2 (see *Underlying data*) ^26^.

### Nucleotide binding proteins are enriched for the presence of LCRs in their C-terminal region

**Figure 5.**
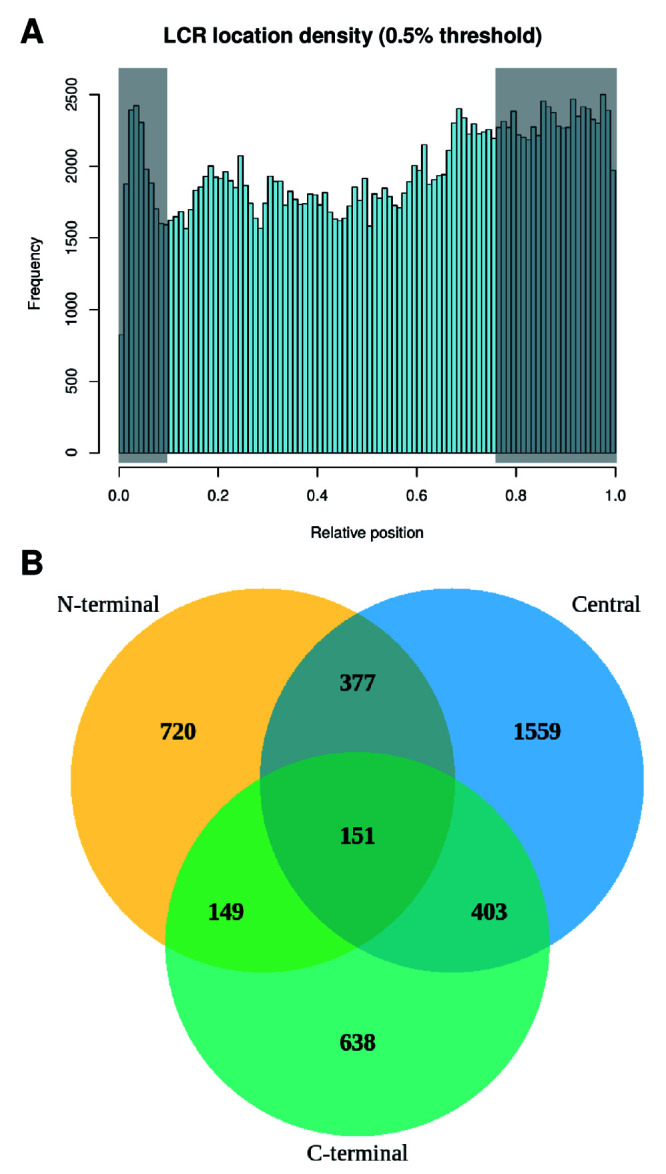
Relative location of low-complexity regions (LCRs) on proteins. **A**) Each position on the proteins, relative to the size of the protein (normalised for each protein to 1, the first 10%, i.e. from 0 to 0.1, and last 25%, i.e. from 0.75 to 1, are highlighted by grey areas), have been analysed for the presence of an LCR. The density of the presence of an LCR has been plotted relative to the size of the proteins for the entire proteome with a threshold of 0.5%. **B**) The Venn diagram represents the number of proteins with at least one LCR (threshold 0.5%): starting and ending in the first 25% of their relative size (yellow: N-terminal); starting and ending between 25%-75% of their relative size (blue: Central); starting after 75% and ending after 80% of their relative size (green: C-terminal). Overlap regions indicate proteins possessing LCRs in two or more of the regions.

Previous studies of LCRs have suggested that the position of LCRs in a protein can influence its function. Coletta *et al.* demonstrated that LCRs in the proteome of *Saccharomyces cerevisiae* were preferentially located toward sequence extremities and that proteins with LCRs at these positions have more binding partners than proteins with LCRs in a more central position ^25^. To analyse the distribution of LCRs in the *Trypanosoma brucei* proteome, we computed the frequency of an LCR for each relative position for all proteins. We excluded VSGs from further analysis which could introduce bias for the characterisation of LCRs for the rest of the proteome. Across the proteome, LCRs were enriched in the amino-terminal 10% and in the last 25% forming the C-terminal regions (Highlighted in Figure 5A by the grey areas).

**Figure 6.**
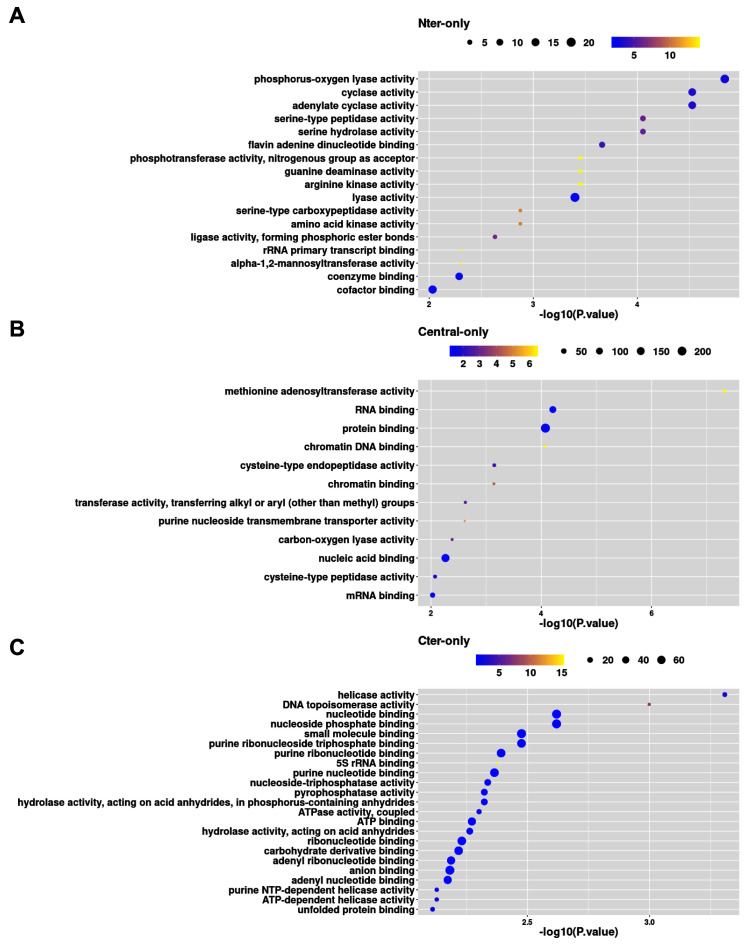
Graphical representation of the -log10 of the p-value obtained with the molecular function Gene Ontology (GO) term analysis for proteins that possess low-complexity regions (LCRs) either only located in their N-terminal ( **A**), Central ( **B**) or C-terminal ( **C**) part. Gradient indicate the log2 fold-change and the size of the dots represent the numbers of proteins.

Reflecting the positional distribution of LCRs, we artificially split the dataset into three categories for proteins containing at least one LCR within the first 25% of the relative protein size (N-terminal), between 25–75% (central) and starting between 75% and ending above 80% of the relative protein size (C-terminal) (depicted in the Venn diagram in Figure 5B). The input data comprised proteins having one or more LCR in their N-terminal region (1397 proteins), central region (2490 proteins) or C-terminal region (1315 proteins). Many proteins had an LCR in more than one region, as indicated by the numbers shown in the Venn overlap regions. Conversely, 720 proteins had a predicted single LCR in their N-terminal domain, 1559 a single centrally-located LCR, and 638 proteins a single C-terminal LCR. Molecular function Gene Ontology analysis indicates that proteins with one or more LCRs are generally enriched for a molecular binding function. Functional enrichment was most notable when the LCR was N-terminal or C-terminal ( Figure 6; supplement file 3, *Underlying data* ^26^, with a p-value < 0.01). Indeed, when located on the N-terminal domain, LCRs were enriched for proteins with predicted cyclase (GO:0009975, 3.8-fold change (FC) with respect to all proteins), hydrolase (GO:0016817 and GO:0016818, 1.3 FC), lyase (GO:0016829, 2.38 FC) and phosphotransferase activities (arginine kinase GO:0004054, 7.03 FC). In contrast, when proteins possessed C-terminal LCRs, they were mainly enriched for nucleotide binding (RNA GO:0003729 (1.96 FC), DNA GO:0031490 (5.78 FC), purines GO:0032555 (1.2 FC), adenyl GO:0032559 (1.27 FC)). We also note some enrichment for cytoskeleton binding (2.01, 1.77 and 1.9 FC, GO:0008092, GO:0008017 and GO:0015631), peptidase (2.82 and 2.27 FC, GO:0004197 and GO:0008234) and hydrolase activities (GO:0016817, 1.39 FC) in the C-terminal LCR subset. Examples of known RNA interactors are highlighted in Figure 4. Alba proteins, PAPBs and translation initiation factors have been identified in P-bodies and stress granules in *T. brucei* ^4^. In conclusion, these results implicate a potential role of LCRs in the function or interactions of nucleotide binding proteins in *Trypanosoma brucei* when positioned in the C-terminal region. Indeed, the enrichment was such that the identification of LCRs in the C-terminal region of proteins with no functional annotation may suggest a possible involvement in nucleotide binding.

### LCRs are highly diverse and present a general increase of polar amino acids

The composition of LCRs can be highly divergent and has been shown to play a major role in, for example, protein liquid-liquid phase separation and the formation of membraneless organelles ^5^. Therefore, understanding the molecular composition and physico-chemical properties of LCRs in *T. brucei* could help us to understand the evolution and function of such regions in this organism.

**Figure 7.**
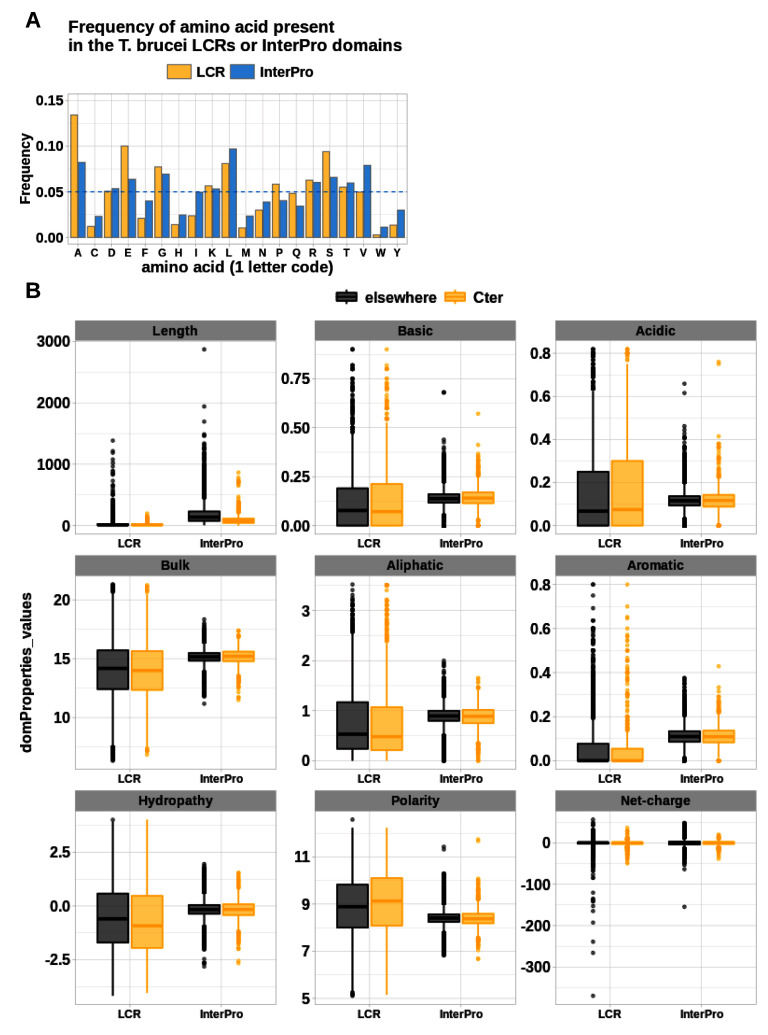
Amino acid composition and properties of low-complexity regions (LCRs). **A**) Frequency of each amino acid in LCRs (yellow) and InterPro domains (blue). Amino acids are indicated with the one letter code and the 1/20th value represented by the blue dashed line to indicate over- or under-representation as an average. **B**) The amino-acid sequence properties (Alkazam R package ^12^) of LCRs and InterPro domains were analysed according to their localisation in the C-terminal region (yellow) or elsewhere (grey) on the proteins. Nine properties were analysed: acid, aliphatic, aromatic, base, bulkiness, net-charge, hydropathy, length and polarity indices.

To start, the relative abundances of the different amino acids were calculated for the identified LCRs and compared to that obtained from domains identified in the InterPro database (TriTrypDB, release 46). The compositional bias of the InterPro domain sequences is highly similar to the total proteome shown in Figure 1 with an enrichment of alanine, glycine, leucine and valine and a poor representation of cysteine, methionine, histidine and tryptophan ( Figure 7A). In contrast, the compositional analysis of LCRs revealed an increase of alanine, glutamine and serine, and a decrease of leucine, proline and valine, relative to the composition observed in the InterPro domains. Contrary to what has been shown in *Plasmodium falciparum* or in yeast prion-like domains, the level of asparagine was relatively low and similar to that observed in the InterPro domain sequence set ^1, 7^.

Several parameters of LCRs have previously been described to influence liquid-liquid phase separation, including LCRs with a polar backbone, punctuated by aromatic and charged amino acids (reviewed in 27, 28). Nine different properties were used to compare InterPro domains and LCRs using the alakazam R package, i.e. the acid, aliphatic, aromatic, basic, bulkiness, net-charge, hydropathy, length and polarity indices ^13^. Comparisons of the domains/LCRs position, whether in the C-terminal region or elsewhere, were then performed for all these properties ( Figure 7B; supplement file 4, *Underlying data* ^26^). The first conclusion from this analysis was that the nature of LCRs is highly diverse compared to defined InterPro domains, and that LCRs are shorter overall. The net charge stays similar between InterPro domains and LCRs (pH7.4), and acid and base indices are only mildly lower in the LCR regions. Interestingly, LCRs are more polar than defined InterPro domains and this is accompanied by a reduction of hydrophobicity ( Figure 7B). There is a reduction of the aliphatic and aromatic indices, also represented by a reduction of bulkiness, indicating an under representation of such amino acids in the highly polar LCRs of the *T. brucei* proteome.

**Figure 8.**
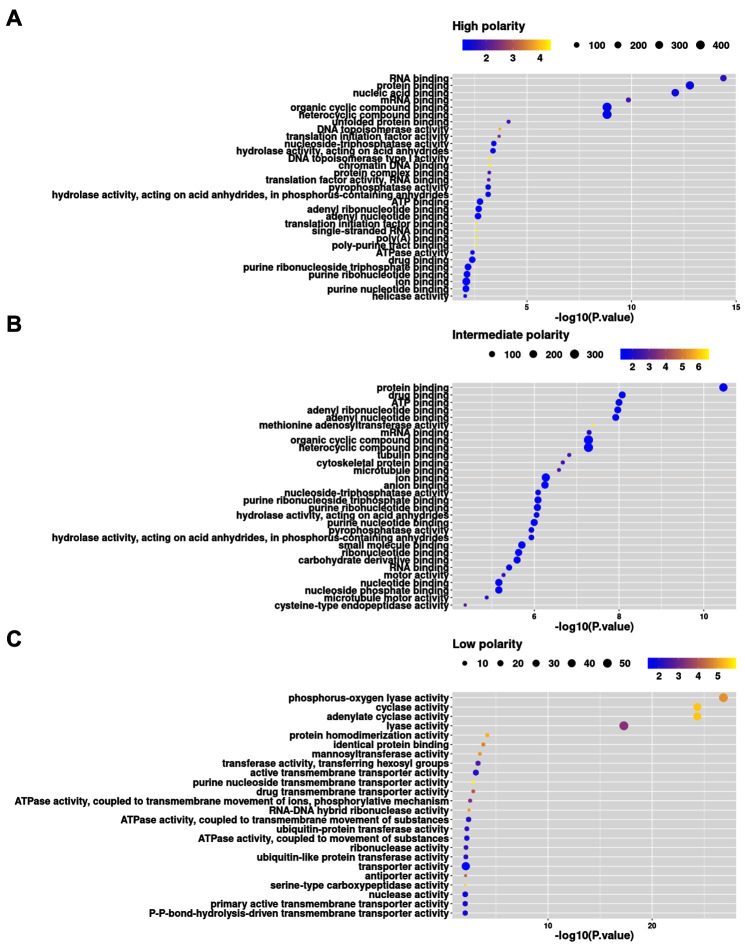
Graphical representation of the -log10 of the p-value obtained with the molecular function Gene Ontology (GO) term analysis for proteins that possess low-complexity regions (LCRs) of either High ( **A**), Intermediate ( **B**) or Low ( **C**) polarity indices. Gradient indicate the log2 fold-change and the size of the dots represent the numbers of proteins.

**Figure 9.**
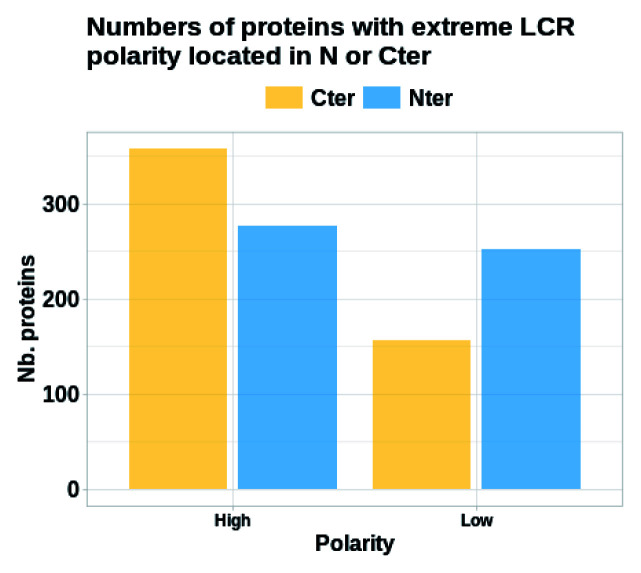
Numbers of proteins with High or Low polarity low-complexity regions (LCRs), located either in the N-terminal or C-terminal.

Due to the diversity of LCRs, we manually subdivided them into three categories, according to their polarity index: below eight (named “low” for the rest of the study), between eight and nine (values where most of the InterPro domains are included, named “intermediate”) and above nine (named “high” for the rest of the study). 2226 proteins have LCRs with high polarity characteristics ( Figure 8; supplement file 5, *Underlying data* ^26^); GO enrichment analysis identified nucleotide binding (RNA, DNA, purine, adenyl, GO:0003723, GO:0003676, GO:0003729, GO:0031490, GO:0032555, GO:0030554) and translation initiation factors (GO:0031369), similar to that observed when considering LCRs located on the C-terminal part of proteins. GO analysis of the 1373 proteins with low polar LCRs showed enrichment for enzymatic activities such as transferase, ATPase, cyclase, lyase and protein transporters, as already noted for N-terminal region LCRs (GO:0016758, GO:0043492, GO:0009975, GO:0016829 and GO:0022804; Figure 8; supplement file 5, *Underlying data* ^26^). Consequently, we compared the list of proteins with extreme LCR polarity to those obtained from the location of LCR at the extremities of the proteins. The majority of proteins with highly polar LCRs had LCRs in their C-terminal region, whereas most proteins with low polar LCRs had LCRs located in their N-terminal extension ( Figure 9; supplement file 5, *Underlying data* ^26^; X-squared = 32.602, df = 1, p-value = 1.131e-8). It can be noted that 1472 genes harbour a signal peptide and one or more LCRs. The overlap between LCRs and signal peptides are presented in supplement file 7 (see *Underlying data*) ^26^.

Overall, these results suggest that highly polar LCRs are located preferentially on the C terminal region of proteins involved in DNA/RNA binding and the regulation of gene expression, whereas low polar LCRs are located mainly on proteins implicated in diverse enzymatic activities. As previously recognised in other organisms, *T. brucei* LCRs are characterised by a reduction of aromatic, aliphatic and basic amino acids, known to enhance liquid-liquid phase separation ^27, 28^.

### LCRs are overrepresented by phosphorylation events in T. brucei

**Figure 10.**
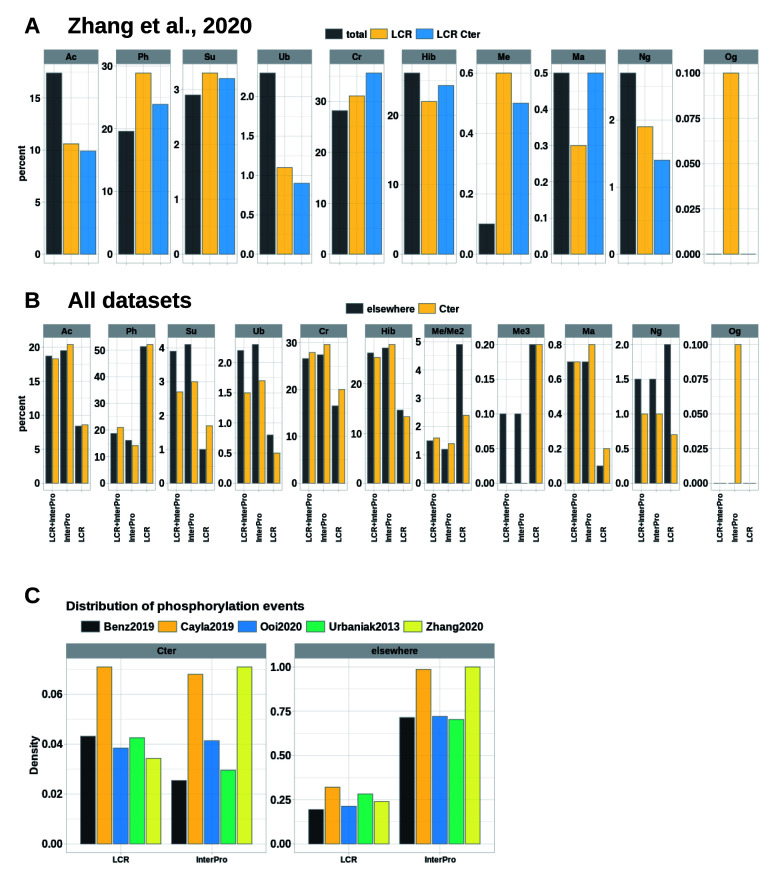
Mapping of post-translational modifications. **A**) The relative representation (percent) of post-translational modifications, identified in *T. brucei* bloodstream parasites by Zhang *et al.* ^15^, were analysed in the total proteome (grey), in the low-complexity regions (LCRs; yellow) or in the LCRs located in the C-terminal region of their corresponding protein (blue). **B**) The Zhang *et al.* dataset ^15^ was merged with those obtained by: Urbaniak *et al.*, 2013, Benz *et al.*, 2019, Cayla *et al.*, 2019, Ooi *et al.*, 2020 for the analysis of phosphorylation, Fisk *et al.*, 2013 and Lott *et al.*, 2013 for the mono/di-methylation and Moretti *et al.*, 2018 for the lysine acetylation ^14– 17, 19– 21, 32^; to obtain the positions of all documented modifications. The relative representation (percent) of post-translational modifications was analysed in the InterPro domains + LCRs, InterPro domains only or LCRs only, according to the position within the domain/LCR on which they are located, i.e. C-terminal (yellow) or elsewhere (grey). The distributions of phospho-residues were compared between the different datasets for their position within domains/LCRs, according to the position of the domain/LCR on which they are located: C-terminal or elsewhere. **C**) Density distribution of the phosphorylation event from the different datasets.

The dynamism of membraneless granule formation, via liquid-liquid phase separation, has been shown to be regulated by post-translational modifications (PTMs) ^29, 30^. Consequently, we looked for the presence of PTMs in the LCRs of the *T. brucei* proteome. First, we analysed the extensive dataset of PTMs of *T. brucei* bloodstream forms obtained by Zhang *et al.* ^15^. We plotted the percentage of each modification relative to the total number of PTMs either independently of their localisation, present in LCRs or present in LCRs located in the C-terminal regions ( Figure 10A; supplement file 6, *Underlying data* ^26^). Among the 10 PTMs analysed in this study, acetylations were decreased in LCRs compared to the whole proteome, as were ubiquitinations and, to a lesser extent, N-glycosylation. In contrast, phosphorylation events were relatively enriched in the bloodstream stage in LCRs independently of the LCR’s localisation within a protein (FC = 1.47, p-value < 0.001).

**Figure 11.**
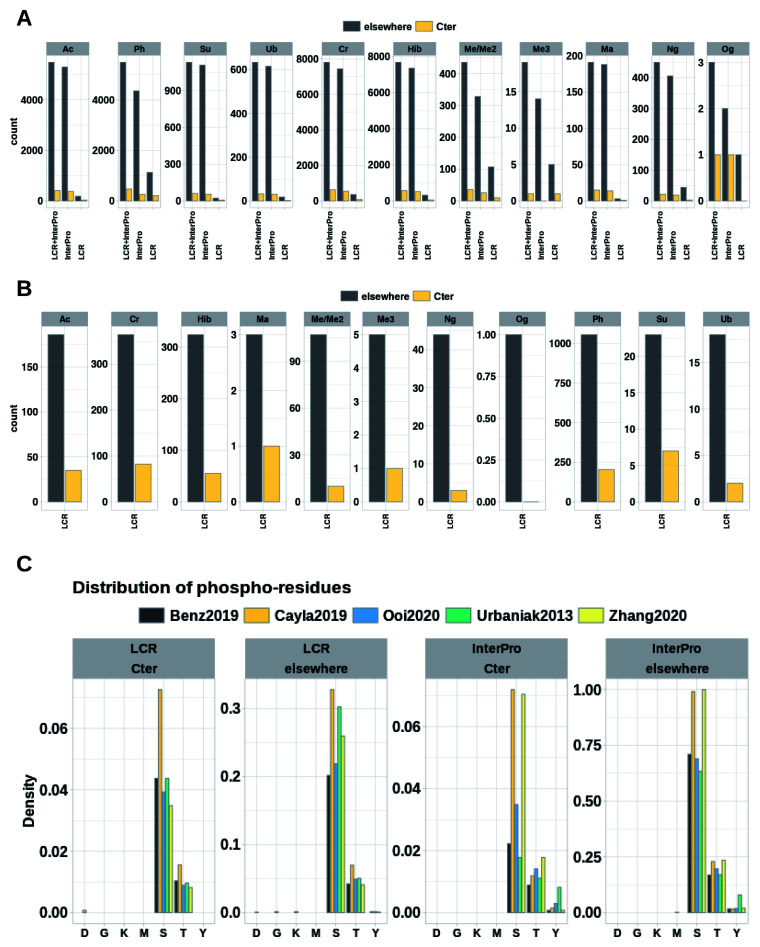
Mapping of post-translational modifications. Raw numbers of PTMs present on ‘LCR and InterPro’, ‘InterPro only’ or ‘LCR only’ ( **A**). The same dataset is plotted on **B**, but only the ‘LCR’ is presented. **C**) Density distribution of the phosphorylation on the residues indicated in X-axis, from the different datasets. PTM, post-translational modification; LCR, low-complexity region.

To have a broader picture of the different possible post-translational modifications, we then merged the dataset of Zhang *et al.* ^15^ with the phosphorylation datasets obtained by Urbaniak *et al.* ^31^, Benz *et al.* ^14^, Cayla *et al.* ^17^, Ooi *et al.* ^32^, the mono/di-methylation datasets obtained by Fisk *et al.* ^21^ and Lott *et al.* ^20^ and also the lysine acetylation dataset obtained by Moretti *et al.* ^19^. It should be noted that we chose to disregard the life cycle stage, stress conditions or the genetically modified strain in which the PTMs were determined. We plotted the percentage of each modification relative to the total number of PTMs in the InterPro domains and LCRs, InterPro domains only or LCRs only, by either looking for the presence of these PTMs in domains/LCRs located in the C-terminal region or elsewhere ( Figure 10B). The raw count numbers of PTMs present on LCRs and InterPro domains are provided in Figure 11 and supplement file 6 (see *Underlying data* ^26^). The combined dataset indicated that LCRs may be relatively depleted of acetylations (FC = 2.31, p-value < 0.001), crotonylations (FC = 1.62, p-value < 0.001) and 2-hydroxybutyrylations (FC = 1.88, p-value < 0.001), with no significant difference between LCRs located in the C-terminal or elsewhere. The same observation was also noted for sumoylations (FC = 3.48, p-value < 0.001) and ubiquitinations (FC = 2.91, p-value < 0.001), whereas an enrichment was observed in methylations (FC = 3.66, p-value < 0.001) in the LCRs. Interestingly, phosphorylations were found to represent ~51% of the modifications observed in LCRs but only ~16% of the modifications observed in the InterPro domains (FC = 3.22, p-value < 0.001, Figure 10B). As this strong enrichment for phosphorylation was less evident in the Zhang dataset, we controlled for bias in the additional datasets by analysing phosphorylations within LCRs. The results presented in Figure 10C indicate a similar distribution of phosphorylation events between all the datasets. Likewise, the distribution of phosphorylation on the different residues is similar between the different datasets ( Figure 11C). We conclude that the relative increase of phosphorylation events in the LCRs is not due to a bias of the datasets analysed but is of likely biological relevance.

**Figure 12.**
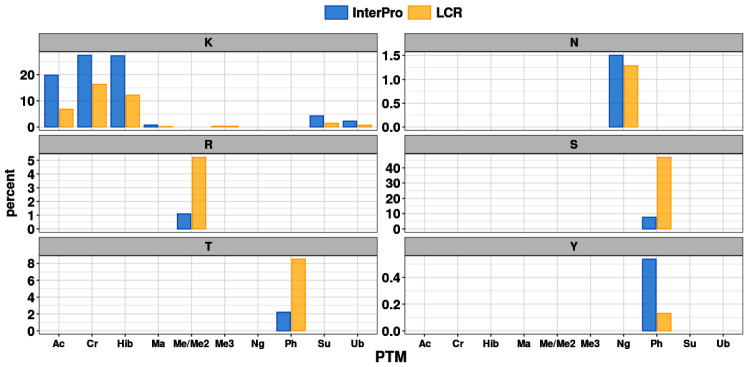
Mapping of post-translational modifications on the corresponding residue. The relative representation (percent) of post-translational modifications was analysed in the InterPro domains (blue) or LCRs (yellow), after normalisation by the frequency of each amino acid in the corresponding regions/domains.

To investigate if the enrichment of phosphorylation events in LCRs was due to the relative increase of phosphorylable residues in these regions, we normalised the percentage of presence of each PTM by the frequency of the amino acid they have been identified on, either for the LCRs or the domains identified in the InterPro database ( Figure 12). The results confirmed our previous observations, with a very strong increase of phosphorylation on LCRs, mainly on serine residues, compared to the InterPro domains.

We finally analysed the dataset published by Lueong *et al*. 2016 ^33^, revealing a set of 155 mRNA-binding proteins, and extracted their LCRs and PTMs. Among these mRNA-binders, 99 proteins harboured one or more LCRs, with 42 having a LCR located in their C-terminal region. 82 of 155 were phosphorylated and 35 were phosphorylated on LCRs, including Alba 4, pumilio/PUF 6 and 9, UBP2 and the zinc finger proteins ZC3H1-like and ZC3H40, for example (supplement file 6, *Underlying data* ^26^). This analysis confirmed and highlighted our observations that nucleotide binding proteins were enriched for the presence of LCRs, particularly in their C-terminal regions, and potentially regulated by phosphorylation.

## Discussion

In this study, we provide a comprehensive analysis of LCRs predicted within the *T. brucei* proteome. A number of the physicochemical properties of LCRs in trypanosomes and the positional biases of LCRs for certain protein classes are likely to be relevant for their biological interactions. Our analysis has revealed the presence of LCRs on 42% of proteins, excluding the VSG repertoire. This indicates that *T. brucei* harbours among the highest level of LCRs in eukaryotes (where 10–20% of proteins have LCRs), similar to other protozoan eukaryotes, *P. falciparum* and *Dictyostelium discoideum* (which each have at least 50%) ^7, 34^.

In yeast, the positions of LCRs in proteins can be a marker for proteins exhibiting enhanced protein interactions when they are located on the extremities of the proteins ^25^. In *T. brucei*, enrichment is similarly observed for a subset of molecular functions, such as enzymatic transferases or nucleotide binding, in the N-terminal and C-terminal regions, respectively. By analogy, the human DYRK3 kinase associates with stress granules via an N-terminal LCR that regulates the granule’s dynamics ^35^; ribonucleotide binding proteins have also been shown to be rich in C-terminal LCRs. Indeed, P-bodies and stress granules, which are membraneless organelles, contain RNA binding proteins enriched for LCRs and depleted for regions with high levels of hydrophobicity (bulky, aromatic and hydrophobic residues) ^36^.

The composition of LCRs and their physico-chemical properties are starting to be understood. For example, yeast proteins containing prion-like domains exhibit a prevalence of polar amino acids and in particular, asparagine, within their LCRs. The same observation has been made in the LCRs of *P. faciparum,* while other species of Plasmodium do not exhibit such properties ^7^. From our analysis, it would appear that in *T. brucei,* LCRs have evolved differently to *P. falciparum*. Indeed, asparagine is an underrepresented amino acid in the proteome and is not enriched in LCRs. However, there is a notable over-representation of two other polar amino acids in the LCRs of *T. brucei*: serine and glutamine. This particular characteristic could suggest that granular structures in *T. brucei* could be ‘harder’ than in other species, as these two residues have been shown to promote hardening through formation of labile-cross-beta-sheets, while glycine enhances fluidity (reviewed in ^28^). The same observation was made for the enrichment of serine in human LCRs ^27^.

Several parameters, intrinsic to the sequence of LCRs, influence phase separation. *T. brucei* LCRs are enriched in polar residues but aromatic residues are under-represented. This confirms previous observations in which LCRs with a polar backbone, punctuated by aromatic and charged amino acids, enhanced protein condensation (reviewed in 27, 28). Our results also suggest that the molecular functions of proteins could influence the nature of the different LCRs in *T. brucei*, or conversely, proteins with enzymatic functions have low polar index LCRs, while proteins involved in nucleotide binding and gene expression regulation have LCRs with a high polar index.

Recent studies have demonstrated that phase separation mediated via LCRs was also a mechanism regulated by post-translational modifications. For example, O-linked-N-acetylglucosamine-glycosylation enhances stress granule formation by favouring aggregation of untranslated messenger ribonucleoproteins (reviewed in 29). It has also been shown that threonine and arginine govern saturation/concentration of phase separation via threonine-threonine interaction, electrostatic interaction (negatively charged amino acids) and threonine-arginine interactions ^28^. These two residues are subject to modification by phosphorylation and methylation, respectively. Arginine methylation of the repetitive RGG or RG motifs present on ribonucleotide binding proteins, reduces liquid-liquid phase separation by interfering with arginine-aromatic interactions (reviewed in 29, 30). Interestingly, in the datasets we analysed, methylations were infrequent, despite their relative enrichment in LCRs. However, there was a marked enrichment of phosphorylation sites in the LCRs of *T. brucei* compared to the rest of the proteome. Phosphorylation modifies the aromatic-cationic interactions or aromatic-aromatic interactions of proteins, which can influence phase separation of ribonucleotide-binding proteins either positively or negatively (reviewed in 29). In the literature, there are now numerous examples of the phosphorylation of residues present on LCRs or adjacent to LCRs that influence phase separation (reviewed in 30). Firstly, phosphorylation on multiple S/T sites on the neurodegeneration-linked protein FUS interferes with phase separation and reduces the binding of the FUS/LCR. This was also shown to have consequences for tethered proteins, which do not possess LCRs, which were less associated with the hydrogel structures when FUS was phosphorylated. A second example is the MARK2 kinase which phosphorylates Tau protein on serine residues in the microtubule associated domain. Tau is an RNA-binding protein that condenses *in vitro* and promotes microtubule polymerisation. The phosphorylation provides additional negative charges which promotes electrostatic interactions and drives phase separation of Tau. Thirdly, in yeast, Ime2 kinase phosphorylates the amyloid-like translational repressor Rim4 on residues located in LCR, causing the de-condensation of Rim4 and its rapid degradation (reviewed in 30).

There are numerous examples of the dynamic formation of stress granules in these and related parasites during nutritional stress ^4, 37– 40^. Recent evidence for altered phosphorylation of RNA regulators has also been observed under conditions of heat stress ^32^. In that study, the authors revealed that nearly 200 sites exhibit changes in phosphorylation on RBPs, protein kinases, translational components, and P- body / stress granule proteins after one hour of heat shock ^32^. Our analysis highlights that 50 of these phosphorylation changes, on 21 proteins, are present on LCRs including on kinases, nucleoporins, ligases and translation initiation factors (eIF4G4, eIF4E3; supplement file 6, *Underlying data* ^26^). In addition, using a published dataset of confidently identified mRNA-binding proteins ^33^, we revealed that 99 proteins out of 155 present LCRs, with 35 proteins phosphorylated on these LCRs, including for example the Alba 4 protein (supplement file 6, *Underlying data* ^26^), previously identified as a component of stress granules in *T.brucei* ^40^. These results reveal potential components implicated in stress granules regulation by phosphorylation. However, it is well known that starvation stress granules and heat shock stress granules ^4, 37– 40^ are compositionally distinct, and we hypothesise that protein targeting to membraneless granules could be regulated by different signalling pathways in response to different physiological stresses.

In conclusion, we propose that the different properties of LCRs (polarity and distribution within resident proteins) and their potential regulation by phosphorylation in *T. brucei* could help to regulate the formation of membraneless granules or the hydrogel microenvironment. Added to this, the local depletion of ATP by active protein kinases targeted to the granular structures or liquid droplets may influence the dynamics of phase separation, as suggested by the study of *Xenopus laevis* oocytes, in which the nucleolus becomes more viscous when ATP is depleted ^41^. In combination, the phosphorylation of LCRs on target proteins and the ATP balance within the microenvironment of the granule could drive the dynamic assembly and disaggregation of gene regulators, controlling the parasite’s adaption to environmental change.

## Data availability

### Underlying data

Zenodo: Cayla *et al.*, 2020 Wellcome Open Research – Underlying data. https://doi.org/10.5281/zenodo.4135199 ^26^.

This project contains the following underlying data:

- **supplement_file_2.xlsx** (Position of every InterPro domain and LCR identified. All genes are provided with indication on chromosome localisation, presence of transmembrane domains, signal peptides and the localisation of the encoded proteins, either predicted using DeepLoc ^42^ or observed (Tryptag ^43^). LCR identified with the SEG algorithm, using the window size of 12, 25, 45 amino acids, are indicated in the third sheet).
- **supplement_file_3.xlsx** (List of genes and Molecular Function gene ontology (GO) enrichment analysis of proteins with predicted LCRs in the N-terminal, central part or C-terminal or the different possible combinations.)
- **supplement_file_4.xlsx** (Property analysis of sequences of every InterPro and LCRs identified.)
- **supplement_file_5.xlsx** (List of genes and Molecular GO enrichment analysis of proteins presenting a Low (<8) or High (>9) polarity index level.)
- **supplement_file_6.xlsx** (List and position of PTMs present on InterPro domains and LCRs. The different datasets from which the PTMs have been extracted can be found in the Zhang2020, Benz2019, Cayla2019, Urbaniak2013, Ooi2020, Fisk2012, Lott2012 and Moretti2017 ^14, 15, 17, 19– 21, 31, 32^ columns. The sequence properties of the domains/LCRs on which these PTMs are located are also indicated. The list of modifications identified in Ooi *et al.* 2020 ^32^ present on LCRs are indicated in the second sheet. Third sheet indicate the list of proteins identified in Lueong *et al.* 2020 ^33^, presenting LCRs and the fourth sheet indicate the PTMs identified in the proteins identified in Lueong *et al.* 2020 ^33^.)
- **supplement_file_7.xlsx** (List and positions of LCRs, signal peptides and their overlapping regions.)

### Extended data

Zenodo: Cayla *et al.*, 2020 Wellcome Open Research – Extended data. https://doi.org/10.5281/zenodo.4135190 ^24^.

This project contains the following extended data:

- **Supplement Figure S1** (Cumulative distribution functions of the entropy values. Representation of the empirical cumulative distribution functions (ecdf) of the entropy values of the *T. brucei* proteome, calculated with the Shannon’s formula as implemented in the entropy.plugin() function, for the different window sizes. The vertical lines represent the different possible thresholds: 0.5, 1, 1.5, 2, 3.5, 4, 4.5, 5% under which LCR have been called.)
- **Supplement Figure S2** (Statistics on the LCRs obtained from different thresholds.Statistical values obtained from the cumulative ecdf distributions for each window size (Windows). Values = numbers of LCRs identified, Mean and SD = mean and standard-deviation obtained from the cumulative ecdf, the remainder of the numbers are the different possible thresholds: 0.5, 1, 1.5, 2, 3.5, 4, 4.5, 5% under which LCR have been called, with their value indicated for each window size.)
- **Supplement** Figure S3 (Comparison of the LCRs obtained the SEG algorithm with three different initial window parameters, 12, 25 or 45 amino acids. A) Distribution of unique amino acids per low complexity region (LCR) after merging. B) Distribution of the length of the LCRs, zoomed to include only those in the range from 0 to 100 amino acids. C) Distribution of the numbers of LCRs per protein, zoomed to include only those in the range from 0 to 10 LCRs per protein. D) Values for different LCR parameters obtained from the 12, 25 and 45 amino acids analysis windows. E) The Venn diagram represents the number of proteins with at least one LCR identified with the SEG algorithm with the windows parameters of 12 amino acids (blue), 25 amino acids (green) or 45 amino acids (red) and the ones identified with the entropy methodology threshold 0.5% (yellow). Overlap regions indicate proteins possessing LCRs with different methodology.)
- **supplement_file_1.pdf** (Visualisation of LCRs, InterPro domain (InterPro) and PTMs for every protein (excluding VSGs) of the *T. brucei* proteome. Each plot represents a protein (ID and product). The X-axis indicates the protein size in amino acids and on the plot are represented the final combined LCRs (in red), the identified InterPro domains in blue and the overlap regions between LCR and InterPro domain indicated in yellow. Post-translational modifications (PTMs) identified in experimental analysis by different studies are in dictated above by “+” symbol. Each modification is coloured in blue when present in an InterPro domain, in red when present in an LCR or in black if present in neither.)

Zenodo: Cayla *et al.*, 2020, Wellcome Open Research - Code availability. https://doi.org/10.5281/zenodo.4135175 ^12^.

This project contains the following extended data:

- **LCR_TREU927_RSCRIPTS_v2.tar.gz** (Compressed file containing the necessary code to generate LCRs of the proteome of *Trypanosoma brucei*.)

Data are available under the terms of the Creative Commons Attribution 4.0 International license (CC-BY 4.0).
