# Supplementary material for: A global analysis of low-complexity regions in the Trypanosoma brucei proteome reveals enrichment in the C-terminus of nucleic acid binding proteins providing potential targets of phosphorylation
Source: Wellcome Open Res. 2020 Nov 18;5:219. Originally published 2020 Sep 21. [Version 2] doi: 10.12688/wellcomeopenres.16286.2 (PMC7682498; doi:10.12688/wellcomeopenres.16286.2)
Supplement: Peer review history

# Peer review: Reviewer response for version 2

**DOI:** 10.21956/wellcomeopenres.18062.r41489

**Published:** 2020-11-19

**Authors:** Susanne Kramer

**Affiliations:** 1 Zell- und Entwicklungsbiologie, Universität Würzburg, Würzburg, Germany

I’m happy with the changes. All my concerns were addressed.

Is the work clearly and accurately presented and does it cite the current literature?

Yes

If applicable, is the statistical analysis and its interpretation appropriate?

Yes

Are all the source data underlying the results available to ensure full reproducibility?

Yes

Is the study design appropriate and is the work technically sound?

Yes

Are the conclusions drawn adequately supported by the results?

Yes

Are sufficient details of methods and analysis provided to allow replication by others?

Yes

Reviewer Expertise:

RNA biology of kinetoplastida, RNA granules, phase separation

I confirm that I have read this submission and believe that I have an appropriate level of expertise to confirm that it is of an acceptable scientific standard.

# Peer review: Reviewer response for version 1

**DOI:** 10.21956/wellcomeopenres.17894.r40535

**Published:** 2020-10-12

**Authors:** Sebastian Hutchinson

**Affiliations:** 1 Trypanosome Cell Biology Unit and INSERM U1201, Institut Pasteur, Paris, France

This manuscript describes the identification of protein low complexity regions by Shannon's entropy method in *Trypanosoma brucei,* and the bioinformatic characterisation of those regions. The authors find low complexity regions in approximately half of the proteome. Instances of polar LCRs are overrepresented in N and C termini of proteins, and in nucleic acid binding proteins. Finally, by comparing LCRs to know post translational modifications the authors find that LCRs are frequently associated with phosphorylation events. This is a useful analysis which I believe will offer new lines of inquiry for the *T. brucei *community. 

 Minor points:

 As the authors state that in the abstract that the purpose of the study was to "generate a substantially more comprehensive genome-wide survey of LCRs", I feel it would be useful to discuss their data in comparison with what is available on TriTrypDB.

 Referencing: specific instances below (I think the appropriate references are in the text, just missing from these statements): 

-  Introduction "By inference from what is known for other model eukaryotes, it is plausible that the aggregation into membraneless structures could be influenced by the presence and/or distribution of LCRs in the protein sequences themselves." 
- Results "The composition of LCRs can be highly divergent and has been shown to play a major role in, for example, protein liquid-liquid phase separation and the formation of membraneless organelles." 

Please specify TriTrypDB versions. I noticed that the R script gave an error as the "current version" used in the script is not the "current" one.

 In running the authors R script, I noticed several Variant surface glycoproteins in the datasets. VSG encoding genes seem to have a variety of annotations on TriTryp. "variant surface protein, putative" and "Variant Surface Glycoprotein, putative" (upper case sensitivity in grep seems to be the reason). 

 Supplement file 1 is reported in the text associated to  http://www.doi.org/10.5281/zenodo.4015044 but is at http://www.doi.org/10.5281/zenodo.4015084 ^1^.

Is the work clearly and accurately presented and does it cite the current literature?

Yes

If applicable, is the statistical analysis and its interpretation appropriate?

Yes

Are all the source data underlying the results available to ensure full reproducibility?

Yes

Is the study design appropriate and is the work technically sound?

Yes

Are the conclusions drawn adequately supported by the results?

Yes

Are sufficient details of methods and analysis provided to allow replication by others?

Yes

Reviewer Expertise:

Trypanosoma brucei molecular biology and bioinformatic analysis

I confirm that I have read this submission and believe that I have an appropriate level of expertise to confirm that it is of an acceptable scientific standard.

## References

[1] : Cayla et al., 2020, Wellcome Open Research - Underlying data.2020; 10.5281/zenodo.4015043 10.5281/zenodo.4015043 Reference source

# Response

**Published:** 2020-10-27

**Authors:** Mathieu Cayla

**Affiliations:** University of Edinburgh, UK

**Reviewer Report 12 Oct 2020**

**Sebastian Hutchinson Trypanosome Cell Biology Unit and INSERM U1201, Institut Pasteur, Paris, France**

 This manuscript describes the identification of protein low complexity regions by Shannon's entropy method in Trypanosoma brucei, and the bioinformatic characterisation of those regions. The authors find low complexity regions in approximately half of the proteome. Instances of polar LCRs are overrepresented in N and C termini of proteins, and in nucleic acid binding proteins. Finally, by comparing LCRs to know post translational modifications the authors find that LCRs are frequently associated with phosphorylation events. This is a useful analysis which I believe will offer new lines of inquiry for the T. brucei community.

 Minor points:

 As the authors state that in the abstract that the purpose of the study was to "generate a substantially more comprehensive genome-wide survey of LCRs", I feel it would be useful to discuss their data in comparison with what is available on TriTrypDB.

- We thank the reviewer for this comment. A discussion comparing the results obtained with SEG algorithm and the entropy methodology has now been added in the paper P8L17-33 and supplement figure S3 and the data were included in supplement file 2.

Referencing: specific instances below (I think the appropriate references are in the text, just missing from these statements): 

1. Introduction "By inference from what is known for other model eukaryotes, it is plausible that the aggregation into membraneless structures could be influenced by the presence and/or distribution of LCRs in the protein sequences themselves."
2. Results "The composition of LCRs can be highly divergent and has been shown to play a major role in, for example, protein liquid-liquid phase separation and the formation of membraneless organelles." 

- Done P6L13 and P11L4.

Please specify TriTrypDB versions. I noticed that the R script gave an error as the "current version" used in the script is not the "current" one.

- As specified in the material and method section – ‘InterPro domain mapping’, the version used at the time of the analysis was the release 46. We have now also added that specification in the LCR identification section P4L30.
- Concerning the code giving an error as “the current version”, althoughthat the reviewer is strictly correct, a comment in the script specifies that the link would need to be changed according to the organism and the date of analysis:

## Download the proteome and interpro datasets

 # NOTE:: wget file link names will depend on the organism and date of download

 In running the authors R script, I noticed several Variant surface glycoproteins in the datasets. VSG encoding genes seem to have a variety of annotations on TriTryp. "variant surface protein, putative" and "Variant Surface Glycoprotein, putative" (upper case sensitivity in grep seems to be the reason). 

- We thank the reviewer for spotting that error. A new version of the script has been uploaded in the code availability section. However, the 59 variant surface genes that were left, were filtered out for downstream analysis and therefore do not affect the results obtained.

Supplement file 1 is reported in the text associated to http://www.doi.org/10.5281/zenodo.4015044 but is at http://www.doi.org/10.5281/zenodo.4015084.

- We thank the reviewer for highlighting that mistake, generated during the editorial processing.

# Peer review: Reviewer response for version 1

**DOI:** 10.21956/wellcomeopenres.17894.r40536

**Published:** 2020-10-06

**Authors:** Susanne Kramer

**Affiliations:** 1 Zell- und Entwicklungsbiologie, Universität Würzburg, Würzburg, Germany

In this study, the authors have used the Shannon’s entropy method to define low complexity regions in the proteome of Trypanosoma brucei. They find LCRs in 42% of all proteins, and have done extensive bioinformatic studies to find common features among the proteins with LCRs in their N or C termini or central region. They used a range of published data on PTMs to investigate negative and positive enrichments among their LCRs. This study is well done and useful, given the current progress in understanding liquid liquid phase separation as a novel contributor to cellular organisation. I only have a few comments.

 The abstract is very general and would benefit from some numbers (how many proteins with LCRs, how enriched are the phosphorylations). 

 The authors show the distribution of amino acid frequency for trypanosomes. How does this compare with other protozoa and opisthokonts; in other words, how unique is this pattern to trypanosomes?

 A discussion on how these new LCR data compare with the currently available LCR annotations with the SEG algorithm is missing. The new LCR data should be integrated into TriTrypDB.

 Go term analysis: I felt these automatic predictions should be presented in a little less raw version. Some can be pooled (in particular features with very few proteins are highly error prone in this kind of analysis) and others can be omitted (Go-features like ‘binding’ or ‘molecular function’ are not very meaningful). I m also not sure whether the p-value is the best way to sort the data, at least the fold enrichment (which is more intuitive) should be shown in addition. RNA binding proteins: As this is a major (and highly interesting) point of this paper, it would be very interesting to explore at least one experimental dataset too, for example the oligo dT binders ^1^. 

 Posttranslational modifications: For all of these positive and negative enrichments, were the differences in amino acid frequencies between LCR and whole proteome considered? In other words, can part of the enrichment in phosphorylation in the LCRs be explained by the fact, that these simply contain a higher proportion of serine residues? (and respectively for all the other PTMs).

Is the work clearly and accurately presented and does it cite the current literature?

Yes

If applicable, is the statistical analysis and its interpretation appropriate?

Yes

Are all the source data underlying the results available to ensure full reproducibility?

Yes

Is the study design appropriate and is the work technically sound?

Yes

Are the conclusions drawn adequately supported by the results?

Yes

Are sufficient details of methods and analysis provided to allow replication by others?

Yes

Reviewer Expertise:

RNA biology of kinetoplastida, RNA granules, phase separation

I confirm that I have read this submission and believe that I have an appropriate level of expertise to confirm that it is of an acceptable scientific standard, however I have significant reservations, as outlined above.

## References

[1] : Gene expression regulatory networks in Trypanosoma brucei: insights into the role of the mRNA-binding proteome. *Mol Microbiol*.100(3) : 10.1111/mmi.13328 457-71 10.1111/mmi.13328 26784394

# Response

**Published:** 2020-10-27

**Authors:** Mathieu Cayla

**Affiliations:** University of Edinburgh, UK

**Reviewer Report 06 Oct 2020**

**Susanne Kramer Zell- und Entwicklungsbiologie, Universität Würzburg, Würzburg, Germany**

 In this study, the authors have used the Shannon’s entropy method to define low complexity regions in the proteome of Trypanosoma brucei. They find LCRs in 42% of all proteins, and have done extensive bioinformatic studies to find common features among the proteins with LCRs in their N or C termini or central region. They used a range of published data on PTMs to investigate negative and positive enrichments among their LCRs. This study is well done and useful, given the current progress in understanding liquid liquid phase separation as a novel contributor to cellular organisation. I only have a few comments.

 The abstract is very general and would benefit from some numbers (how many proteins with LCRs, how enriched are the phosphorylations). 

- The abstract has now been updated to include numbers, as suggested P3.

The authors show the distribution of amino acid frequency for trypanosomes. How does this compare with other protozoa and opisthokonts; in other words, how unique is this pattern to trypanosomes?

- As indicated in the text, LCRs of *Trypanosoma brucei* are not enriched in asparagine. This observation differs from what has been seen in *Plasmodium falciparum* and yeast prion-like domains. However, this enrichment in asparagine has not been observed in other Plasmodium species.

The under representation of this polar amino acid could be compensated, in the LCRs of *T. brucei*, by the over-representation of serine and glutamine residues. The over-representation of serines in LCRs was already described in Humans (Martin and Mittag, Biochemistry, 2018). The serine enrichment, as well as the presence of glutamine is thought to influence the fluidity of the aggregates by promoting hardening through the formation of labile-cross-beta-sheets.

 These comparisons with other organisms, highlighting the unique features of LCRs of *T. brucei*, are present in the discussion.

 A discussion on how these new LCR data compare with the currently available LCR annotations with the SEG algorithm is missing. The new LCR data should be integrated into TriTrypDB.

- We thank the reviewer for this comment. A discussion comparing the current SEG data to the entropy analysis has now been included in the Results section P8L17-33 and supplement figure S3 and the data were included in supplement file 2.
- We also agree with the reviewer that these data should be included in the TriTrypDB and have initiated dialogue with the database curators to do so.

Go term analysis: I felt these automatic predictions should be presented in a little less raw version. Some can be pooled (in particular features with very few proteins are highly error prone in this kind of analysis) and others can be omitted (Go-features like ‘binding’ or ‘molecular function’ are not very meaningful). I m also not sure whether the p-value is the best way to sort the data, at least the fold enrichment (which is more intuitive) should be shown in addition. RNA binding proteins: As this is a major (and highly interesting) point of this paper, it would be very interesting to explore at least one experimental dataset too, for example the oligo dT binders.

- Regarding the Go term analysis, we have now updated the Figures 6 and 8 to include the representation of the fold changes (gradient) and the number of proteins used for each molecular function (size of the dots), as suggested by the reviewer. The generic terms “binding” and “molecular function” have also been filtered out. However, we believe that ordering the data by p-value is the most appropriate way since this reflects the significance of the value unlike fold change, Therefore, we did not modify this representation.
- We thank the reviewer for the suggestion of using the mRNA-binding proteins dataset published by Lueong et al. In this dataset, 155 proteins have been confidently identified as mRNA binding proteins. 99 proteins possess a LCR with 42 of them having a LCR in their C-terminal region. 82 of these proteins are phosphorylated with 35 on an LCR. This analysis confirms our observation that RNA binding proteins are rich in LCR. This discussion point has been added in the text P15L5-12 and P16L41-45 and results have been included in supplement file 6.

 

 Posttranslational modifications: For all of these positive and negative enrichments, were the differences in amino acid frequencies between LCR and whole proteome considered? In other words, can part of the enrichment in phosphorylation in the LCRs be explained by the fact, that these simply contain a higher proportion of serine residues? (and respectively for all the other PTMs).

- The reviewer raised a very interesting question. To look if the relative abundance of phosphorylable residues in LCRs (mainly serine) compared to the rest of the proteome could explain the enrichment of phosphorylation in these regions, we normalised the percentage of each post-translational modifications by the relative frequency of the residue on which they have been identified. The results can now be found in Figure 12 and P14L33-P15L4. Briefly, no change is observed after normalisation by the frequency of the corresponding residue, confirming our observations that phosphorylation events are enriched in LCRs.
